# Continuous Conversion of Cellulose and Lignin-Cellulose Precursors to Carbon Fibers: The Importance of Stretching during Low-Temperature Carbonization

**DOI:** 10.1021/acsomega.6c06352

**Published:** 2026-10-02

**Authors:** Andreas Bengtsson, Mijung Cho, Jenny Bengtsson, Maria Sedin, Helena Westerback, Michael Hummel

**Affiliations:** † Division Bioeconomy and Health, RISE Research Institutes of Sweden, Stockholm 114 28, Sweden; ‡ Department of Bioproducts and Biosystems, 174277 Aalto University, Espoo 02150, Finland; § Division Material and Production, 11248 RISE Research Institutes of Sweden, Mölndal 431 53, Sweden; ∥ Department of Chemistry and Chemical Engineering, Chalmers University of Technology, Gothenburg 412 96, Sweden

## Abstract

Cellulose-lignin-based fibrous carbon materials have received significant attention as sustainable alternatives to petroleum-derived carbon fibers. While prior studies have mainly focused on spinning parameters and thermal processing to enhance mechanical performance, limited research has addressed the influence of stretching these biocomponent filaments during carbonization on molecular alignment, crystalline structure, and resulting mechanical properties. In this study, continuous cellulose-lignin mixed filaments containing 0, 30, and 50% lignin were produced using a dry-jet wet spinning method. The filaments underwent tailored stabilization and carbonization using a lab-scale continuous line, with varying draw ratios (DR = 1–1.5) applied during carbonization at 850 °C. The effects of stretching and lignin content on the carbon fibers’ properties were evaluated through tensile testing, wide-angle X-ray scattering (WAXS), and Raman spectroscopy. Lignin-containing filaments demonstrated improved stretchability compared to pure cellulose fibers. Especially, carbon fibers derived from precursor fibers with 50 wt % lignin showed a 25% increase in Young’s modulus under DR = 1.5 (49 GPa) relative to DR = 1 (41 GPa). After high-temperature carbonization at 1100 °C, the modulus further increased to 58 GPa compared to 46 GPa for unstretched fibers, corresponding to an enhanced molecular orientation factor (0.6 vs 0.5) confirmed by WAXS. This approach provides a promising route toward higher performance of bioderived carbon fibers.

## Graphical abstract

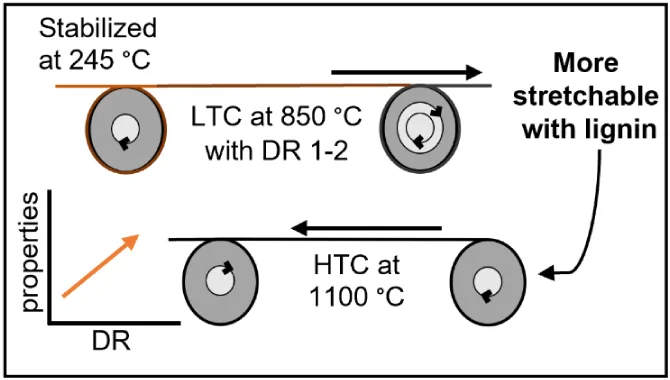

## Introduction

The global production of carbon fibers (CFs) has increased steadily during the last 15 years (with the exception of 2020) and reached 187 kt in 2024, corresponding to an annual growth of 13%.^1^ The production is propelled by the surging demand from the wind energy sector and a strong growth of commercial airplane production, as air traffic has recovered after the COVID-19 pandemic. Currently, CF applications are roughly split into 50% for industrial end uses, 30% for aerospace, and 20% for sporting goods.

CFs have a carbon content of 92–100 wt % and very high specific strength and modulus, with commercial CFs reaching strengths of 2.5 to 7 GPa, making them suitable as reinforcing fibers in lightweight composites.^2^ The most common polymer used for CFs is polyacrylonitrile (PAN), which is a nonrenewable and expensive substrate. The relatively high costs of CFs have thus far limited their use to the three application sectors mentioned above. Thus, more cost-efficient and renewable raw materials are sought after to reach broader markets and new applications.

At the same time, the pulping industry is undergoing a significant transition toward biorefineries. Pulping processes are redesigned to allow for the separation and valorization of other wood constituents like hemicelluloses and lignin. Processes to separate kraft lignin from the black liquor of a Kraft pulp mill are already established.^3,4^ Lignin and cellulose have individually been used as raw materials for CFs. Already in the 1960s, lignin, offering an intrinsic carbon content of ≥60%, was investigated as CF precursor material. Lignin with a sufficiently low molar mass shows a thermoplastic character and can be melt-spun into continuous filaments.^5^ However, the macromolecular structure and properties of lignin vary depending on the wood species from which it is derived and the process used to isolate it.^6,7^ Consequently, not every type of lignin qualifies for melt spinning. Often, other polymers are needed as plasticizers,^8,9^ or chemical modification of the lignin substrate is used to alter the glass transition temperature.^10^ Most importantly, though, melt-spun filaments require extensive thermal stabilization before carbonization to prevent the filaments from melting and fusing.^11^

Pure cellulose filaments have also shown limitations in their potential as a CF precursor. Although it was possible to produce CFs with tensile strengths of 1–4 GPa and moduli of 170–690 GPa from viscose filaments when graphitized at 2500 °C (Thornel series),^12,13^ any cellulosic precursor suffers from a low carbon yield upon carbonization. Various volatile carbonaceous compounds formed during the heat treatment reduce the actual carbon yield far below the theoretical carbon content of 44.4%, sometimes down to 10%.^2,14^ Slow heating rates or carbonization catalysts such as sulfuric and phosphorus acids and salts thereof are used to increase the yield but reduce the economic efficiency of the process.^15–17^

Direct dissolution of cellulose and lignin in a nonderivatizing solvent and spinning both components in a Lyocell-type process has shown great potential to overcome the limitations of pure cellulose and lignin precursors. Classical Lyocell solvents such as *N*-methylmorpholine *N*-oxide (NMMO) monohydrate, but also emerging solvents for lignocellulose such as imidazolium-, amidine-, and guanidine-based ionic liquids (ILs), were reported as suitable for spinning of cellulose-lignin filaments.^18^ The resulting fibers were then converted into biobased CFs.^19^ Bengtsson et al. have used 1-ethyl-3-methylimidazolium acetate to dissolve pulp cellulose and lignin and spin them into precursor fibers.^20^ They then studied the effect of different heating rates during thermostabilization on the properties of the final CFs.^21^ Trogen et al. and Duc et al. used cellulose-lignin filaments spun from 1,5-diazabicyclo[4.3.0]­non-5-ene-1-ium acetate ([DBNH]­OAc) and converted them to CFs using a continuous pilot carbonization line.^22,23^ Catalysts such as phosphates and borates could also increase the char yield in the case of cellulose-lignin precursor fibers.^24^ It is well established that tension and stretching during the stabilization and carbonization process are essential to develop the mechanical properties of the final CFs.^2^ Respective studies have been conducted for cellulose-derived CFs already in their early development phase in the 1970s.^25^ However, only a few studies address this aspect for lignocellulosic precursor filaments, and most of them focus on hot stretching.^26,27^ For instance, stretching of cellulose precursors during low-temperature carbonization (LTC), typically in the 300–900 °C range, improves the mechanical properties of the resulting CFs.^16,27–29^ There is, however, a lack of knowledge on how the addition of lignin affects the stretchability and carbon structure formation. Studies on the effect of stretching during the stabilization process are only indicated in the patent application of Garoff et al.^30^ In the study at hand, we investigate the effect of kraft lignin content on the stretchability of a cellulose-lignin precursor filament during continuous stabilization and then further carbonization. Fibers with different lignin content (0, 30, and 50%) were stretched, and the maximum draw ratio (DR_max_) filament rupture was determined. Specifically, this work focuses on stretching during low-temperature carbonization and its impact on carbon fiber structure and properties.

## Experimental Section

### Materials

Softwood kraft lignin, produced by the LignoBoost process, was obtained from the LignoDemo plant (Bäckhammar, Sweden). The lignin contained 1.2 wt % carbohydrates, 94 wt % Klason lignin, and 6.3 wt % acid-soluble lignin and had an ash content below 1 wt %. The elemental composition of the lignin was 64 wt % C, 25 wt % O, 1.4 wt % S, 5.4 wt % H, and 0.11 wt % N. More details of the lignin can be found in Bengtsson et al. (2018).^20^

Prehydrolyzed kraft (PHK) birch pulp ([η] = 494 mL/g) was received from Stora Enso Enocell mill in Finland. The pulp was received as sheets and ground using a cutting mill (Fritsch, Germany) to a fine powder (<1000 μm).

1,5-Diazabicyclo­[4.3.0]­non-5-ene (DBN) (CAS: 3001-72-7, molar mass = 124.187 g/mol, Fluorochem, UK) and acetic acid (glacial 100%, CAS: 64-19-7, molar mass = 60.05 g/mol, Merck, Germany) were used as received. Tap water, cooled to 9 ± 2 °C, was used in the spin bath. Polydimethylsiloxane (PDMS) emulsion (45% silicon content) was purchased from ThermoFisher.

### Spinning of Precursor Fibers (PFs)

#### Dry-Jet Wet Fiber Spinning

The preparation of IL and dope, as well as fiber spinning, was performed with the Ioncell process as described in previously published methods.^22,31^ The IL 1,5-diazabicyclo[4.3.0]­non-5-enium acetate ([DBNH]­OAc) was prepared following previous work. DBN was mixed with acetic acid in a 6-L glass reactor with a cooling (25 °C) unit. After the slow addition of acetic acid to DBN until a 1:1 molar ratio, the solution was further mixed for an hour at 70 °C with mechanical stirring. The prepared IL was tightly closed and stored at room temperature. Dopes with 0, 30, and 50 wt % lignin with respect to the total polymer concentration were prepared based on previous work in which 30% was considered an optimal trade-off between preserved cellulose crystallinity and increased carbon fiber yield. Lignin and pulp were dissolved in the IL using a vertical kneader equipped with an overhead stirrer at 80 °C for 2 h, mixing at 30 rpm under reduced pressure of 35–40 mbar. The cellulose concentration of the dope was set to about 10 wt % dry solid content. Spinning dopes containing 30 and 50 wt % lignin needed higher total polymer concentrations to maintain suitable rheological properties and ensure stable spinning conditions (see Table 1).^22^ The dissolved cellulose-lignin mixtures (dopes) were filtered with a metal filter (pore size: 6 μm) using an automated filter press at 85 °C to remove undissolved parts or impurities. The filtered dope was dry-jet wet spun using a piston-spinning unit (Fourné Polymertechnik, Germany), with an air gap of 1 cm. A 400-hole spinneret was used with a capillary diameter of 100 μm and L/D 0.02. The extrusion rate in the spinning process was 5.5 cm^3^/min at 70 ± 2 °C. The spin bath consisted of tap water at 10 ± 2 °C. Precursor fibers (PFs) with different cellulose-lignin ratios and various draw ratios (DR) were collected continuously and collected on a bobbin, as shown in Table 1. After spinning, the fibers were washed with tap water using a customized washing line with 20 ± 0.25 min residence time at 65 ± 2 °C. Washed fibers were treated with 0.2 wt % silicon solution in water before air-drying them at 80 °C to enhance fiber handling after heat treatments.^32,33^ Effects of spin finish addition were studied previously,^34^ and PDMS showed the best performance among the investigated surfactants and was therefore adapted in the current study.

**Table 1:** Summary and Sample Code for Precursor Fibers

Pulp content (%)	Lignin content (%)	Dope conc. (%)	DR^a^	Titer (dtex)	Fiber code
100	0	13	4.5	2.73 ± 0.2	L0
70	30	15	6.0	2.53 ± 0.15	L30
50	50	18	6.0	3.06 ± 0.38	L50

a $\mathrm{Draw Ratio}\left(DR\right)=\frac{\mathrm{Take up velocity}}{\mathrm{Extrusion velocity}}$ .

As shown in Table 1, two lignin concentrations were selected based on previous studies to balance spinnability with maximizing carbon fiber yield. Earlier, a lignin content of 30% combined with cellulose showed the optimal balance between fiber performance and yield.^35,36^ At 30% lignin, the crystallinity and molecular orientation of the cellulose matrix were preserved, while the carbon yield increased significantly compared to lower lignin contents.^36^ The maximum amount of lignin that was tolerated for successful wet-jet spinning with the ionic liquid system used herein was determined to be 50%.^22^

The total polymer concentration of each dope was adjusted based on previous studies.^22,23^ Solutions with a higher relative lignin content required an increase in the total polymer concentration to maintain a cellulose concentration that provides the correct viscoelastic properties and allows for stable spinning. Consequently, to arrive at precursor filaments with approximately the same diameter, the solutions had to be spun at different draw ratios (DRs). For the L0 sample, a DR of 4.5 was chosen to obtain high-quality filaments leading to carbon fibers with a good diameter after carbonization and pyrolysis-induced mass loss. A similar diameter was reached for the L30 filament at a DR of 6. L50 would require a slightly higher DR. However, spinning was stable only at DR 6.

### Continuous Stabilization and Carbonization

The PFs were continuously stabilized and carbonized with a setup described in detail in Bengtsson et al.^21,27^ In summary, the setup consisted of a winding system (Xplore Instruments BV, Netherlands), a horizontal tube furnace (OTF 1200x, MTI Corporation, USA), and a yarn tensiometer (ETX Solo, Eltex AB, Sweden). The tube furnace had a total heated length of 0.6 m, with three heating zones of 0.2 m each. To ensure the desired temperature profile, the furnace temperature was calibrated with a thermocouple (CL3515R, Omega Engineering Inc.). For stretching trials, the draw ratio (DR) of the fiber tow was adjusted by varying the relative speed of the unwinder and winder godets. DR = 1 means that the winding godets had the same speed, and the fiber tow was thus prevented from shrinkage, whereas DR > 1 means stretching of the fibers. The stretchability during low-temperature carbonization (LTC, 700–850 °C) was studied as a function of PF composition and conversion conditions used. In this work, stretchability is defined as the DR (DR_max_) at which the fiber tow ruptured. The stretchability trials were performed by increasing the DR with about 0.05 every minute until rupture of the tow. The studied conditions are summarized in Table 2. For preparation of larger amounts of carbonized fiber for material testing, and to ensure a stable conversion without fiber rupture, the stabilized lignin-cellulose PFs were LTC-treated at DR 1.0 and DR 1.5, respectively, whereas the cellulose PFs were LTC-treated at DR 1.0 and DR 1.2–1.4, respectively. High-temperature carbonization (HTC) was done at 1100 °C.

**Table 2:** Summary of the Stabilization and Carbonization Conditions Used in This Study^a^

	Furnace zone temperature (°C)					
Conversion step	Inlet	Middle	Outlet	Gas	DR	Time (min)
Stabilization 1	220	240	245	air	1.00	30 or 60
Stabilization 2	250	260	275	air	1.00	30
**LTC**	500	600	700	nitrogen	1.00–2.40	10
**LTC**	350	600	850	nitrogen	1.00–2.10	10 or 20
**HTC**	1100	1100	1100	nitrogen	0.98	10

a The outlet temperature (zone 3) in each step is used throughout the text to denote the temperature profiles. LTC—low-temperature carbonization; HTC—high-temperature carbonization.

### Characterization

#### Mechanical Testing of Fibers

The tensile properties of the precursor fibers (PFs) were measured using a Favigraph single-fiber tester (Textechno H. Stein GmbH & Co. KG, Germany) according to the standard SFS-EN. The PFs were conditioned at 20 ± 2 °C and 65 ± 2% RH at least overnight before the testing. The following conditions were used for testing: load cell 20 cN, gauge length 20 mm, and test speed 20 mm/min. For each sample, 20 fibers were measured with a pretension weight of 150–200 mg.

Single fiber tensile tests of the carbon fibers (CFs) were performed on an LEX820/LDS0200 (Dia-Stron Ltd., UK) equipped with a laser diffraction system for diameter determination (CERSA-MCI, France). The CFs were tested at 21 ± 2 °C and 31 ± 2% RH using a fixed gauge length of 20 mm at elongation speeds of 5 mm/min and 0.6 mm/min, respectively. UvWin software (Diastron Ltd., UK) was used for date evaluation. The reported values are averages of 30–40 individual CF measurements.

#### Raman Spectroscopy of Carbon Fibers

Raman spectroscopy was performed with a wavelength of laser 532 nm and an 830 l/mm grating, including an optical microscope with a 20× objective (inVia Qontor, Renishaw, Gloucestershire, U.K.) on CF surfaces at three separate positions of three individual filaments per carbon fiber sample. The installed software, WiRE 5.3, is used for instrument setting and analyzing spectra. Ten accumulations per sample at 10% laser power with 1 s exposure time were collected in the range of 222–3748 cm^–1^ centered at 2200 cm^–1^. The spectra were normalized with 0,1 and an average of three spectra per sample using Origin Pro software. The D (∼1336 cm^–1^) and G (∼1592 cm^–1^) bands were fitted by applying a Lorentzian fitting with Origin Pro software. The ratio of two bands, I_D_/I_G_, was calculated as the ratio of the D and G bands’ intensity (I, heights).

#### Scanning Electron Microscopy

The morphology of the precursors and of the CFs was evaluated in an SU3500 scanning electron microscope (Hitachi, Japan) at an acceleration voltage of 3 kV. Prior to imaging, the fibers were sputtered with Au. Cross sections of precursors were prepared by cutting the fibers with a scalpel in liquid nitrogen or using tensile fracture surfaces, while CFs were cut at room temperature.

#### Composition and Structure

The yield of the CFs prepared via continuous conversion was estimated by determining the linear density, measured by weighing 100 cm of fiber tow after each conversion step and dividing that by the linear density of the PFs. The elemental composition (wt %) of the heat-treated fibers and of the CFs was estimated by energy dispersive X-ray analysis (EDXA) using an Xflash detector (Bruker Corp., Billerica, MA, USA) at an acceleration voltage of 15 kV and the Esprit software for data evaluation.

Wide-angle X-ray scattering (WAXS) data of PFs and CFs were collected on an Anton Paar SAXSpoint 2.0 system (Anton Paar, Graz, Austria) equipped with a Microsource X-ray source (Cu K-alpha radiation, wavelength 0.15418 nm) operating at room temperature and a chamber pressure of 1–2 mbar. A Dectris 2D CMOS Eiger R 1M detector with a 75 μm by 75 μm pixel size and a sample-to-detector distance of 111 mm was used. All measurements were performed on fiber tows aligned in a sample holder perpendicular to the beam. The preferred orientation (*f_c_*) of the crystallites was calculated according to eq 1 below:

1 $$f_{c}=\frac{180-\alpha }{180}$$

where α is the full width at half height of the azimuthal profile of the reflection. The signal of the (002) plane of graphite was used for CFs.

## Results and Discussion

The choice of stabilization and LTC conditions was based on earlier experience.^21,22,27^ Initial trials were performed with the L30 precursor using the LTC profile with a final temperature of 700 °C, but due to practical reasons, more focus was later put on the 850 °C profile instead, as the latter profile gave fibers with more distinguishable tensile properties. Figure 1 shows DR_max_ values of the LTC-treated fibers prepared from the different precursors (L0, L30, and L50) using different stabilization conditions. Irrespective of the precursor, DR_max_ was surprisingly high across all samples (DR 1.4–2.4). In particular, the lignin-containing precursors L30 and L50 generally had reached higher DR_max_ than L0, particularly when using the milder stabilization profile (245 °C, 30 min) and when oxidative stabilization was omitted. This could be due to a plasticizing effect of the thermoplastic lignin. After complete stabilization, lignin is converted into a thermoset with no detectable *T*_g_ and thus no softening at higher temperatures, which might explain why the harshest stabilization rendered a lower DR_max_. This is further supported by an experiment where the L30 precursor was stabilized with the 245 °C profile for 60 min instead of 30 min, which led to a reduction of DR_max_ from 2.4 to 2.0 (Table S1). Cellulose being the dominant polymer in the bicomponent fibers certainly affects the plasticity of the precursor fibers too. It was shown earlier that during stabilization cellulose first undergoes fragmentation, decreasing the filament tenacity markedly but also increasing its elongation at break. However, stabilization also induces cross-linking reactions that render the fiber matrix notably stiffer.^17,37^ In contrast to the lignin-containing fibers, the L0 precursor displayed the highest DR_max_ in LTC when using the harshest stabilization profile (275 °C, 30 min), as shown in Figure 1a. Notably, the filament color of L30 and L50 became black regardless of the applied stabilization conditions, whereas L0 was almost unchanged (white) when using the 245 °C profile but became dark brown after using the 275 °C profile.^37^ This is due to the higher degradation temperature of cellulose compared to lignin (Figure S1) and in agreement with earlier studies on the stretchability of cellulose fibers during carbonization.^16^ Overall, this suggests that the stretchability in LTC is strongly influenced by the choice of stabilization conditions and that suitable stabilization conditions differ between a precursor consisting of only cellulose compared to one made of a lignin-cellulose blend.

**Figure 1:**
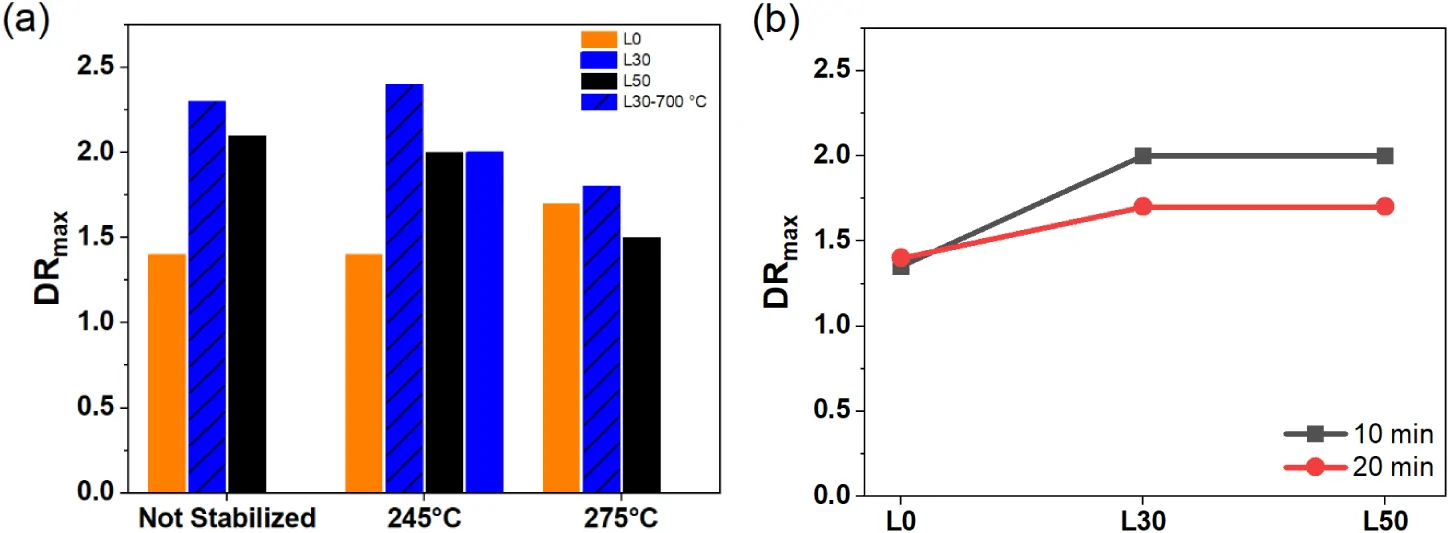
Maximum draw ratio (DR_max_) during LTC of the precursors stabilized at various conditions: (a) DR_max_ as a function of PF composition and stabilization temperature using 10 min LTC residence time; (b) the effect of LTC residence time on DR_max_ using a stabilization temperature of 245 °C. The temperature for LTC was 850 °C except for the blue bar (L30-700 °C) in (a).

To gain more understanding, additional experiments were done by impregnating the L30 precursor with a typical catalyst, ammonium dihydrogen phosphate (ADHP, 0.1 M), which is known to catalyze the dehydration reactions of cellulose during stabilization, potentially affecting DR_max_ during initial heat treatment.^20^ DR_max_ of the ADHP-impregnated L30 precursor was consistently lower than the corresponding fibers without ADHP. For example, stabilization (245 °C, 30 min) and LTC (850 °C, 10 min) of the ADHP-impregnated precursor resulted in a DR_max_ of 1.3, which is significantly lower than when prepared without ADHP (DR_max_ 2.0), which further supports the theory that the stabilization intensity affects the stretchability during LTC. It has been pointed out that cross-links of cellulose may be formed during stabilization, depending on the harshness of this stage, which may affect the stretchability and could potentially explain this behavior.^37^ These results are also in good agreement with the experimental data presented in the patent by Garoff et al.,^30^ who also observed that the stabilization intensity of a lignin-cellulose precursor (50/50 wt/wt, lignin/cellulose) had a strong impact on the resulting stretchability during LTC.

When varying the residence time in the LTC furnace (Figure 1b), no effect could be observed for the L0 precursor. In contrast, both L30 and L50 showed a lower DR_max_ when an LTC residence time of 20 min instead of 10 min was applied, suggesting that the latter condition was preferable for more stretchable fibers during the LTC process. This difference in DR_max_ could be due to a difference in the actual heating rate, since the LTC time was adjusted by altering the speed of the tow using the winding system. The LTC time of 10 min corresponds to a speed of 6 cm/min, whereas the LTC time of 20 min was obtained by using a speed of 3 cm/min, meaning that the heating rate when entering the hot zone of the furnace is different. This behavior can be explained by a previous study on the effect of heating rate on thermo-rheological properties of lignin.^38^ In that work, lignin exposed to a slower heating rate of 1 °C/min showed a lower softening temperature (*T*_g_ = 217 °C) and a lower tan δ peak, whereas a faster heating rate of 5 °C/min led to a higher *T*_g_ (233 °C) and a higher intensity of tan δ peak. Thus, when lignin-containing fibers experienced a relatively fast heating profile (10 min residence time), lignin likely behaved more as a viscous liquid than as an elastic solid. As a result, the shorter residence time made the L30 and L50 fibers more stretchable than those treated with longer residence times. Yet, further experiments are required to confirm this.

The effect of LTC stretching on the fiber properties (850 °C profile) was investigated alongside the evaluation of DR_max_ as described above. A DR lower than DR_max_ was chosen to ensure stable processing, i.e., no tow rupture during 1 h of continuous processing, to obtain sufficient sample material for testing. For L0, stable processing was achieved when DR was 1.2–1.4 (depending on the stabilization profile), but for the L30 and L50 precursors, DR 1.5 was used in all experiments. For comparison, fibers with DR 1.0 (no shrinkage) were prepared to elucidate the effect of LTC stretching. After LTC, the carbon content of the fibers was 87–89 wt % irrespective of conditions used (Table S3). The LTC fibers from L0 had a low gravimetric yield of only about 7 wt %, which increased to 19–21 wt % and 26–30 wt % in the case of L30 and L50, respectively, confirming the beneficial effect of lignin on the yield after carbonization. The carbon yield was not affected by DR in LTC, which suggests that stretching can be used in LTC without additional mass loss. The carbon yield is primarily determined by the chemical composition of fibers and the thermal treatment protocol of stabilization, carbonization, and possibly graphitization. The physical changes that the polymer chains undergo during stretching and the reduction in diameter seem to have very little effect on the residual mass.

The yield of 26–30 wt % for CFs prepared from L50 agrees with earlier reported yields of a lignin-cellulose precursor with the same lignin-cellulose ratio processed at similar conditions.^27^

During manual handling of the CFs, it was noted that the CFs from L50 were easy to handle and separate, whereas those from L0 and L30 were partially fused. Inspection of some CF tows with SEM revealed more severe fiber fusion of L0 and L30 compared to L50, which explains why the CFs from L50 were much easier to handle and separate after LTC (Figure S2). This difference may be attributed to the larger mass loss of L0 and L30 compared to L50, which affects the formation of sticky tars that possibly could recondense on the fiber surfaces (a well-known phenomenon in CF production). The large difference in fiber separability could also be due to a variation in retention of the spin finish PDMS applied during the precursor spinning. PDMS has already been shown to improve fiber separability of an L50 precursor, indicating that PDMS acts as a physical barrier that prevents these individual filaments from sticking together. PDMS typically degrades completely between 300 and 400 °C.^34^ This stability allows PDMS to remain on the fiber surface long enough to protect it through the critical “softening window” of the lignin. Moreover, PDMS might have a slightly higher affinity to lignin-containing fibers than pure cellulose precursors.^36^

The tensile properties of the LTC-derived CFs (Young’s modulus and tensile strength) are presented in Figure 2a–b (a full summary of the properties is given in Table S2). All CFs that were stretched (DR 1.2–1.5) during LTC had a higher Young’s modulus (*P*-value < 0.05) than the corresponding CFs prepared with DR 1.0 (no shrinkage). The modulus for L0, L30, and L50 improved from 41, 36, and 38 GPa to 49, 54, and 49, respectively, due to the internal molecular orientation (Figure 2 and Table S2, Figures S8–S10). The WAXS results showed an increase in the preferred orientation of the 002-plane, in particular for L30 (0.49 to 0.62) and L50 (0.43 to 0.60) (Table S4). The tensile strength decreased slightly, presumably due to an increase in cross-sectional heterogeneity of the CF structure. However, this difference was not statistically significant (*P*-value > 0.05, 0.1–0.9).

**Figure 2:**
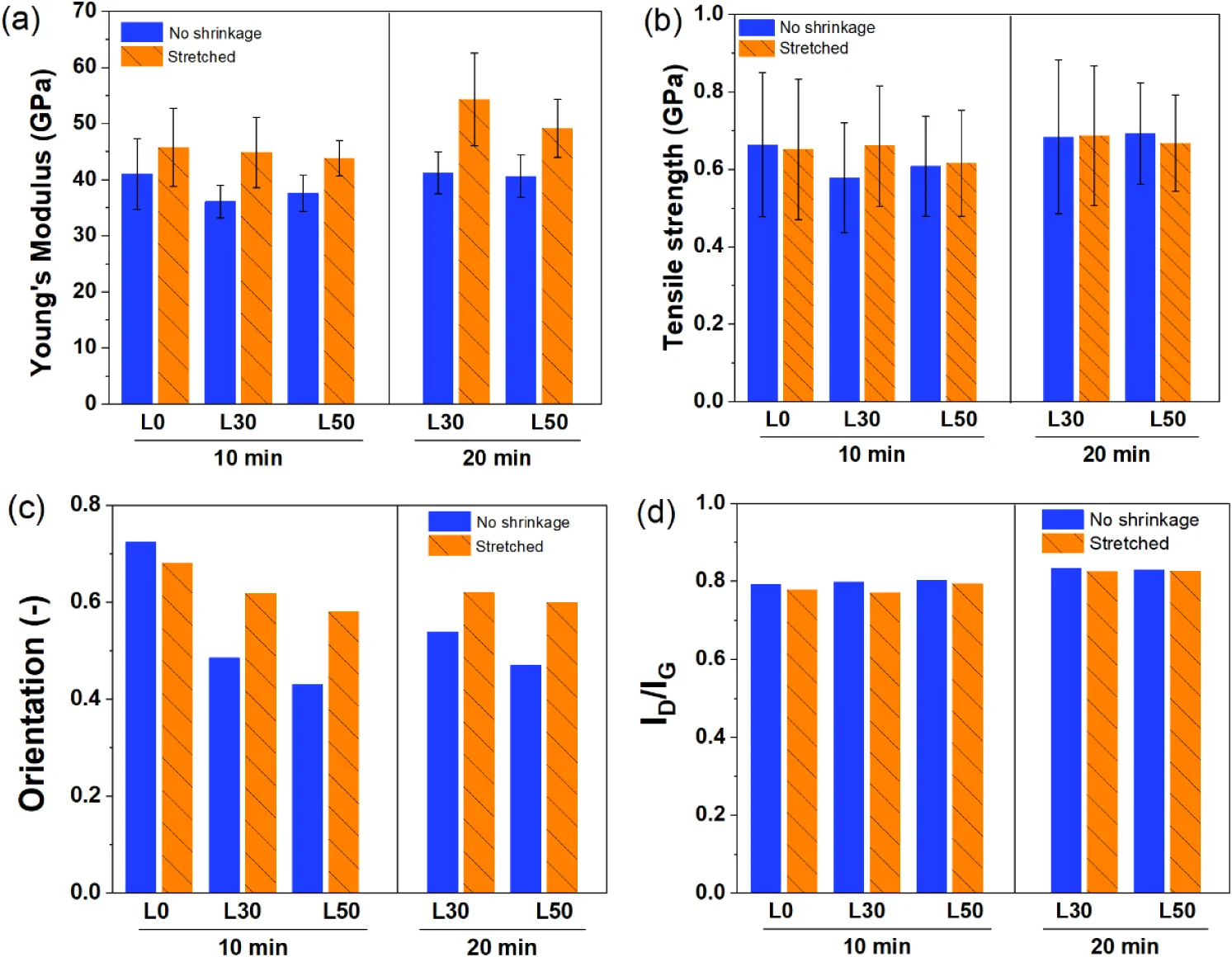
Properties of LTC fibers: (a) Young’s modulus, (b) tensile strength, (c) orientation derived from WAXS measurements, and (d) I_D_/I_G_ in the Raman spectra of LTC fibers as a function of DR, PF composition, and LTC residence time. No shrinkage = DR 1.0; stretched = DR 1.2 (L0) or DR 1.5 (L30 and L50). Stabilization conditions: 245 °C for 30 min. LTC temperature: 850 °C. (Error bars for Young’s modulus and tensile strength represent the standard deviation.).

As shown in previous studies,^21,39^ at temperatures above 1000 °C, significant radial heterogeneity develops within the fiber. This is often caused by insufficient oxygen diffusion into the core during the earlier stabilization stage or different rates of decomposition for heteroatoms in the center versus the surface. This “skin-core” structure creates internal stresses that lower the overall strength. Also, higher heat treatment can lead to the formation of more internal defects or voids. Because carbon fibers are brittle, they follow the “weakest link” theory, where even a small increase in the size or number of defects significantly reduces the stress required to break the fiber.^16^ With decreasing fiber diameter after stretching during low-temperature carbonization as shown in Table S2, the thinner fibers contain a much lower volume per unit length. This reduces the relative presence of structural defects. With lower structural interruptions, stress is distributed evenly across the cross-section, allowing the fiber to achieve its maximum stiffness value.^40^ Samples with an LTC residence time of 10 min did not show any difference in elongation at break when heat-treated with or without stretch. However, there was a significant difference (*P* < 0.05) between the samples without and with stretching after a residence time of 20 min (Table S2).

The WAXS-derived orientation factor is indicative for the molecular orientation of the crystalline domains, with a higher orientation factor indicating crystallites oriented in the fiber direction. The orientation of the crystalline domains predominantly influences the stiffness of the fibers. The correlation with the fibers’ tenacity, however, requires a more differentiated analysis because overall crystallinity and the presence of defects can also affect it. The molecular orientation calculated from the WAXS measurements (Figure 2c, Table S4) shows that the LTC stretching induced a higher anisotropy for the lignin-containing fibers. The increase in molecular orientation explains the higher modulus of the stretched fibers. For the L0 fiber, the already high molecular orientation was not increased by LTC stretching.

All fibers showed a similar I_D_/I_G_ ratio calculated from the Raman spectra (Figure S7a, Figure 2d). This implies that the carbon structure in the fibers is not affected by LTC stretching, perhaps slightly by the LTC residence time.

### Effect of DR during LTC on the HTC Fiber Properties

Based on the LTC results, only the L50 fiber was further carbonized at high temperature (HTC, 1100 °C). For both fibers undergoing no-shrinkage and stretched LTC, an increase in 002 orientation was observed in the WAXS diffractograms (see Figure S6 and Table S4) after the HTC step. The orientation in the 002 direction increased from 0.51 (no-shrinkage) to 0.60 (LTC-stretched). In line with the LTC results, the increase in orientation correlated with an enhanced modulus of the fibers. The Young’s modulus and tensile strength of the L50 fiber increase from 46 GPa and 660 MPa at DR 1 to 58 GPa and 860 MPa at DR 1.5, respectively (Figure 3a and c, and Table S2).

**Figure 3:**
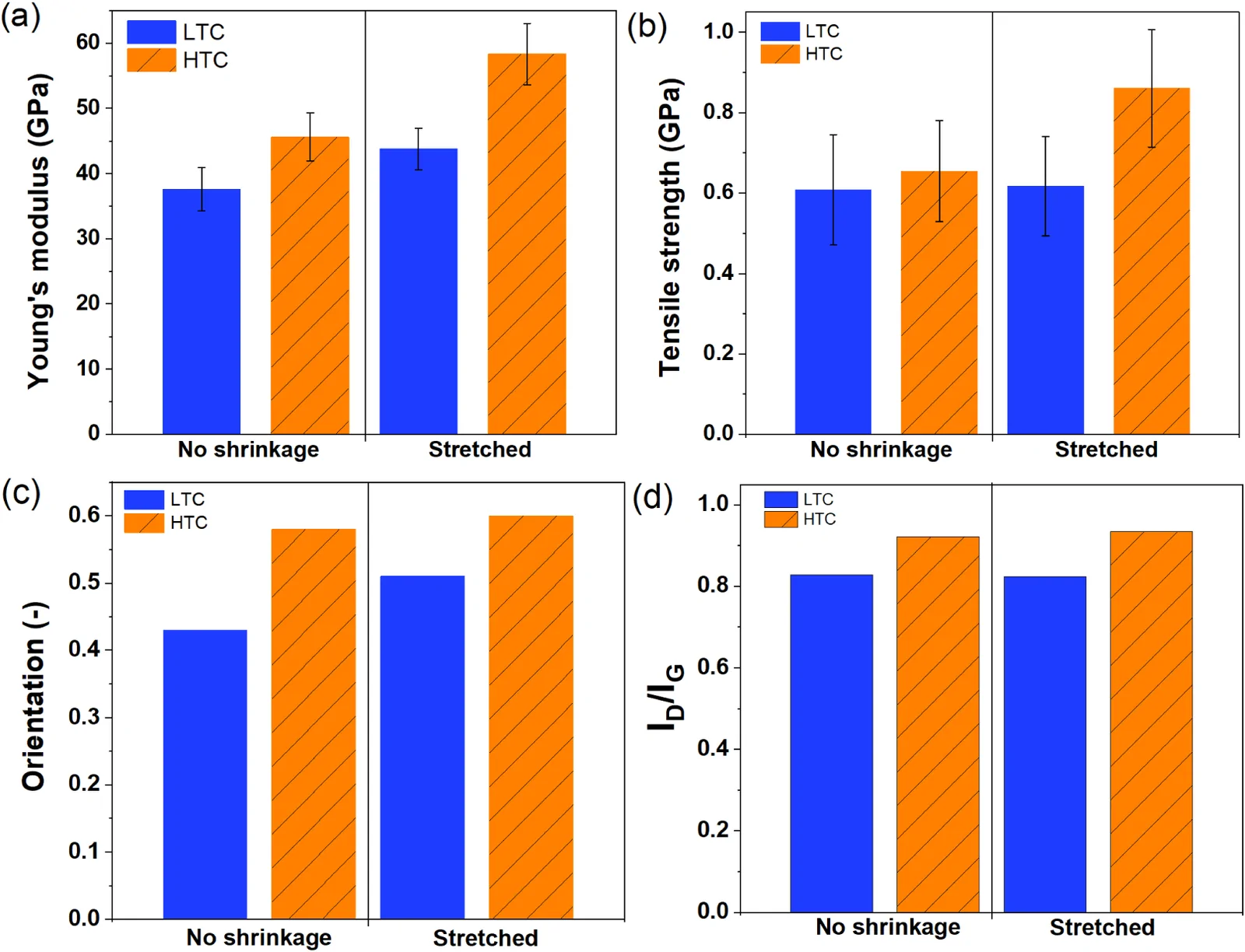
Effect of stretch during LTC on L50-derived CF properties after HTC (1100 °C) treatment: a) Young’s modulus, b) tensile strength, c) orientation (WAXS), and d) I_D_/I_G_-ratio in the Raman spectra. No shrinkage = DR 1.0; stretched = DR 1.5. (Error bars for Young’s modulus and tensile strength represent the standard deviation.).

The HTC treatment increased the I_D_/I_G_ ratio of the no-shrinkage and stretched fiber (Figure S7b), a trend that has been observed previously for carbon fibers prepared at similar temperatures (1100 °C).^32^ Similar to LTC fibers, the stretching during LTC did not influence the I_D_/I_G_ ratio and, thus, did not increase the orientation of the fibers’ carbon matrix during carbonization at 1100 °C. The full-width-at-half-maximum (fwhm) values of the G- and D-bands correlate with changes in the structural order of the carbonized/graphitized fibers. An increased ordering of the carbon matrix results in sharper peaks and smaller fwhm values. Presented in Table S4, the fwhm-D and G values do not show a significant difference between LTC samples with and without stretching. However, the fwhm values for both the D and G bands decreased at 1100 °C from 235 to 177 and from 85 to 75 cm^–1^, respectively. This is in agreement with previous work that showed that upon increase of the carbonization temperature from 600 to 1600 °C, the fwhm of the D and G bands decreased from 157 and 74 to 56 and 51 cm^–1^, respectively.^32^ This indicates improved structural homogeneity of the CFs with increasing carbonization temperature, supporting the gradual transition from amorphous carbon to nanocrystalline graphite.

Interestingly, HTC carbon fibers prepared with stretch during LTC showed an apparent shift toward higher tensile strength compared to the no-shrinkage fibers. The tenacity of carbon fibers is known to be dependent on the orientation and the morphology of the voids and pores inside the fibers. The enhanced tenacity of the stretched fibers can thus be partly explained by the elongation of pores and defects during stretching, leading to an anisotropic pore shape distribution within the fiber. This anisotropy is also observed in the WAXS signal of the LTC-stretched fibers, while the no-stretch fiber shows an entire isotropic profile (see Figure S5). Unterweger et al. also reported higher modulus and average tensile strength for lignin–cellulose-based carbon fibers that had been kept at no shrinkage compared to free shrinkage during LTC, although the increased tensile strength was not statistically significant.^41^ The results demonstrate that stretching during LTC can increase both the tensile modulus and strength of lignin–cellulose-based carbon fibers after HTC. Lastly, there was no difference in the elongation at break between samples with and without stretch during LTC after the final HTC treatment.

Previous investigations on fibers spun from a softwood kraft lignin and kraft pulp blend (70/30) showed that a temperature increase from 1000 to 1600 °C improved the modulus slightly from 67 to 77 GPa but also led to a strength reduction from 1050 to 630 MPa.^32^ Therefore, further studies are needed to evaluate the full potential of cellulose-lignin hybrid fibers as precursors for CFs, in particular when applying industrial equipment and higher final process temperatures.

## Conclusions

Cellulose and lignin-cellulose precursor filaments could be stretched significantly during low-temperature carbonization (LTC) at 700–850 °C. The draw ratio at break, DR_max_, varied from 1.2 to 2.4 depending on the precursor composition and conversion conditions during stabilization and LTC. Lignin-containing precursors were generally more stretchable during LTC than a 100% cellulose precursor. These results suggest that harsher stabilization conditions are positive for the stretchability of cellulose precursors but negative for the lignin-cellulose precursors.

Based on the results of DR_max_, LTC fibers were produced with DR 1.0 and 1.2–1.5. By increasing the DR, the orientation of the carbon structure (002-plane) increased from 0.43–0.60 to 0.58–0.64, and the Young’s modulus rose from 36–41 GPa to 44–54 GPa.

The positive effect of DR on tensile properties and orientation after LTC was also observed during high-temperature carbonization (HTC) at 1100 °C. By increasing the DR during LTC from 1.0 to 1.5, the Young’s modulus increased from 46 to 58 GPa, the tensile strength increased from 660 to 860 MPa, and the orientation of the 002-plane increased from 0.51 to 0.60.

This work intends to pave the way toward a sustainable and scalable method for producing biobased carbon fibers through simple tension-controlled processing during the low-temperature carbonization process. The carbonized fibers exhibited improved mechanical properties without the use of chemicals or excessive energy consumption due to high processing temperatures. This method serves as a guide for large-scale production of biobased carbon fibers. However, further investigations at temperatures above 1100 °C would be needed to gain a more comprehensive understanding of the effect of LTC-stretching on the resulting HTC processability and final CF properties, respectively.

## Supplementary Material



## Notes

Thermogravimetric analysis of fibers and raw materials; SEM images, WAXS plots, and Raman spectra of the carbon fibers; tables summarizing the yield, composition, and mechanical and structural properties of the carbon fibers (PDF)

## Abbreviations

ADHP: ammonium dihydrogen phosphate
CF: carbon fiber
[DBNH]­OAc: 1,5-diazabicyclo­[4.3.0]­non-5-enium acetate
DR: draw ratio
HTC: high-temperature carbonization
LTC: low-temperature carbonization
PDMS: polydimethylsiloxane
