# Supplementary material for: Continuous Conversion of Cellulose and Lignin-Cellulose Precursors to Carbon Fibers: The Importance of Stretching during Low-Temperature Carbonization
Source: ACS Omega. 2026 Oct 2;11(40):61335–43. doi: 10.1021/acsomega.6c06352 (PMC13670868; doi:10.1021/acsomega.6c06352)
Supplement: Supplementary file 1 [file ao6c06352_si_001.pdf]

Supporting information

Continuous conversion of cellulose and lignin-cellulose precursors to carbon fibers: The importance of stretching during low-temperature carbonization

Andreas Bengtsson[1], Mijung Cho[2], Jenny Bengtsson[3,4], Maria Sedin[1], Helena Westerback[2] and Michael Hummel[2]*

[1]Division Bioeconomy and Health, RISE Research Institutes of Sweden, Stockholm, Sweden;

[2]Department of Bioproducts and Biosystems, Aalto University, Espoo, Finland

[3]Division Material and Production, RISE Research Institutes of Sweden, Mölndal, Sweden

[4]Department of Chemistry and Chemical Engineering, Chalmers University of Technology, Gothenburg, Sweden

Thermogravimetric analysis (TGA)

The thermal stability of fiber was measured using a Netzsch STA449 F3 Jupiter & QMS 403 Aeolos Quadro thermal analyzer with a nitrogen flow of 70 ml/min. The 10-15 mg of sample was placed in the alumina crucible, and the mass changes were recorded at 40 to 800 °C, with a heating rate of 10 °C/min. The curves were analyzed with NETZSCH Proteus Thermal Analysis 8.0.

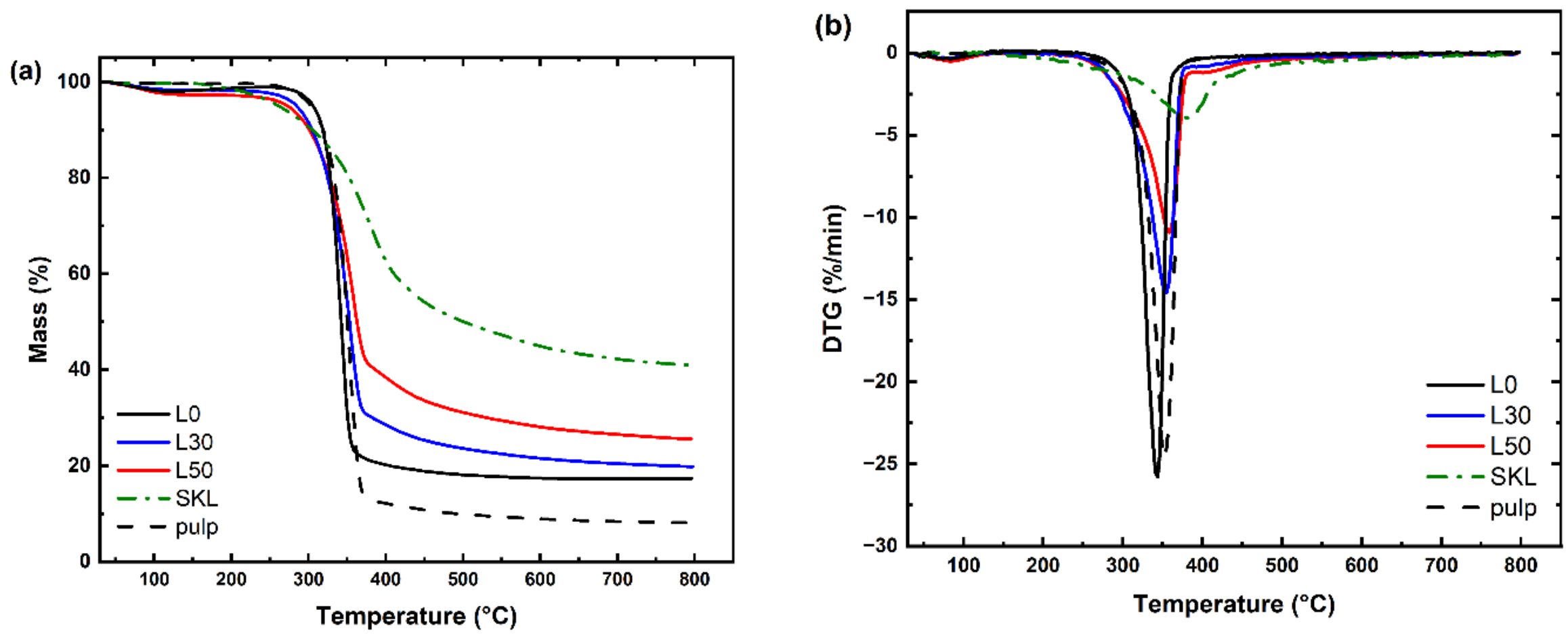

Figure S1. (a) TGA and (b) DTG curves of lignin (SKL), cellulose pulp, and precursor fibers with different lignin content.

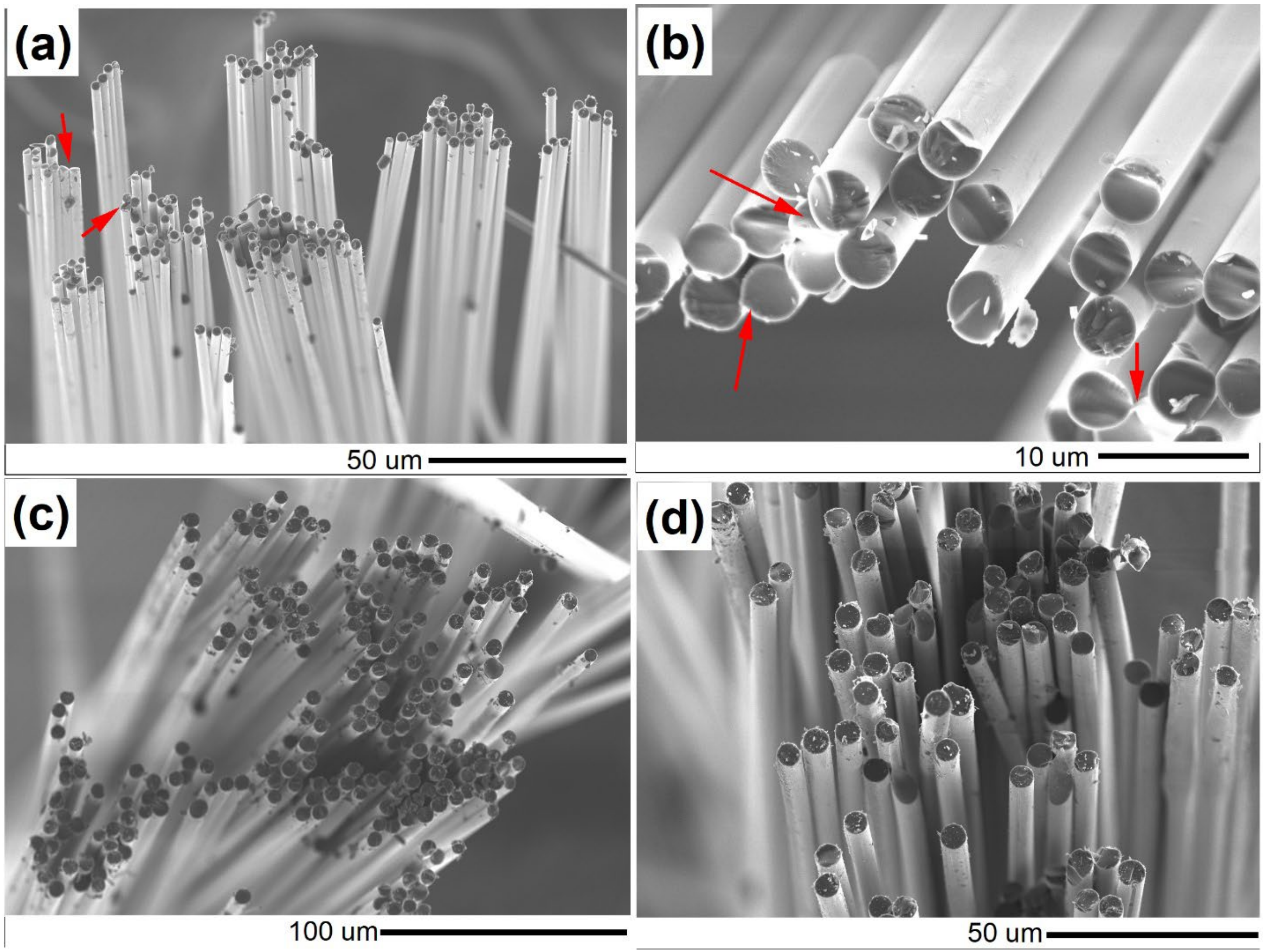

Figure S2. SEM images of carbon fibers from (a) L0 (DR 1.0) at LTC, (b) L30 (DR 1.5) at LTC, (c) L50 (DR 1.5) at LTC and (d) L50 (DR 1.5) at HTC. The precursor filaments were stabilized at 245 °C for 30 min before LTC treatment at 850 °C for 10 min.

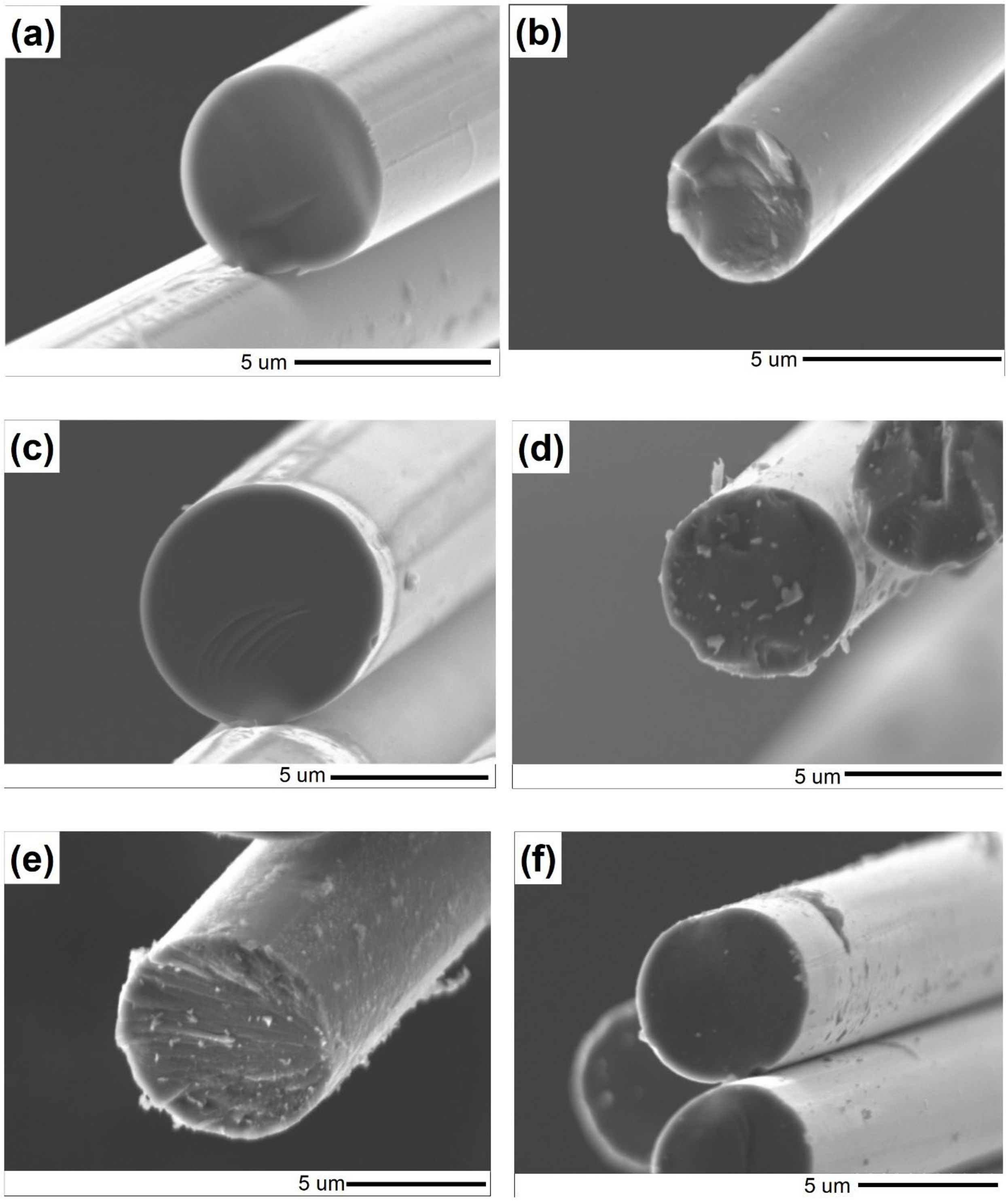

Figure S3. SEM images of cross section of carbon fibers from (a) L30 (DR1) at LTC, (b) L30 (DR1.5) at LTC, (c) L50 (DR1) at LTC, (d) L50 (DR1.5) at LTC, (e) L50 (DR1) at HTC (f) L50 (DR1.5) at HTC

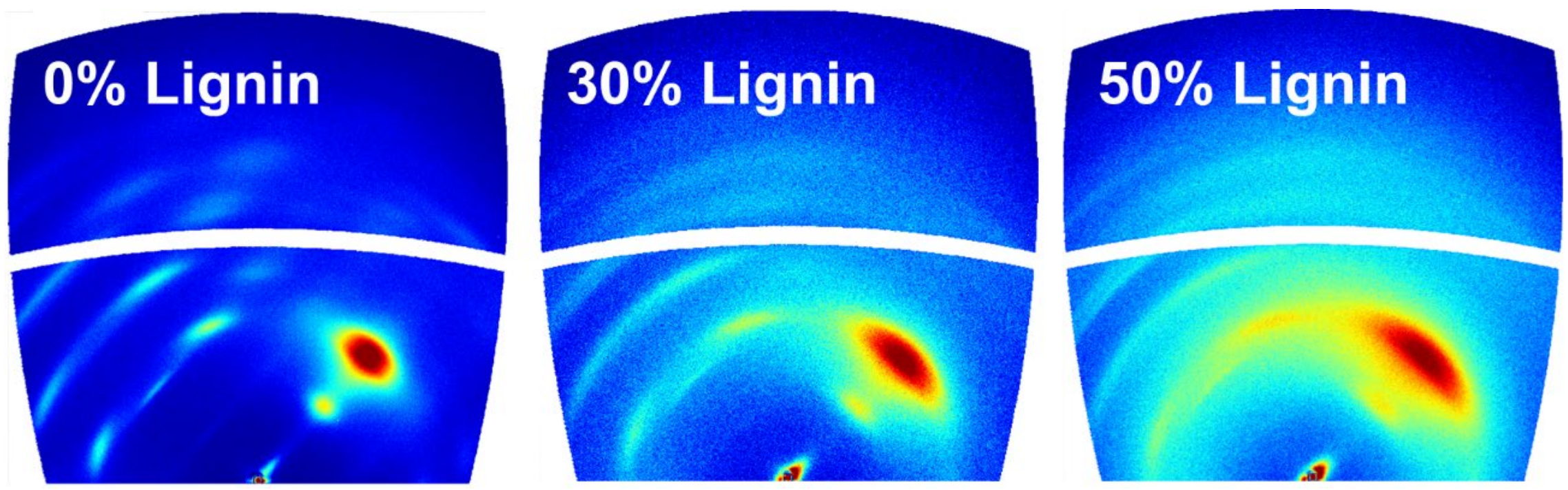

Figure S4. WAXS 2D images of precursor filaments having different lignin content.

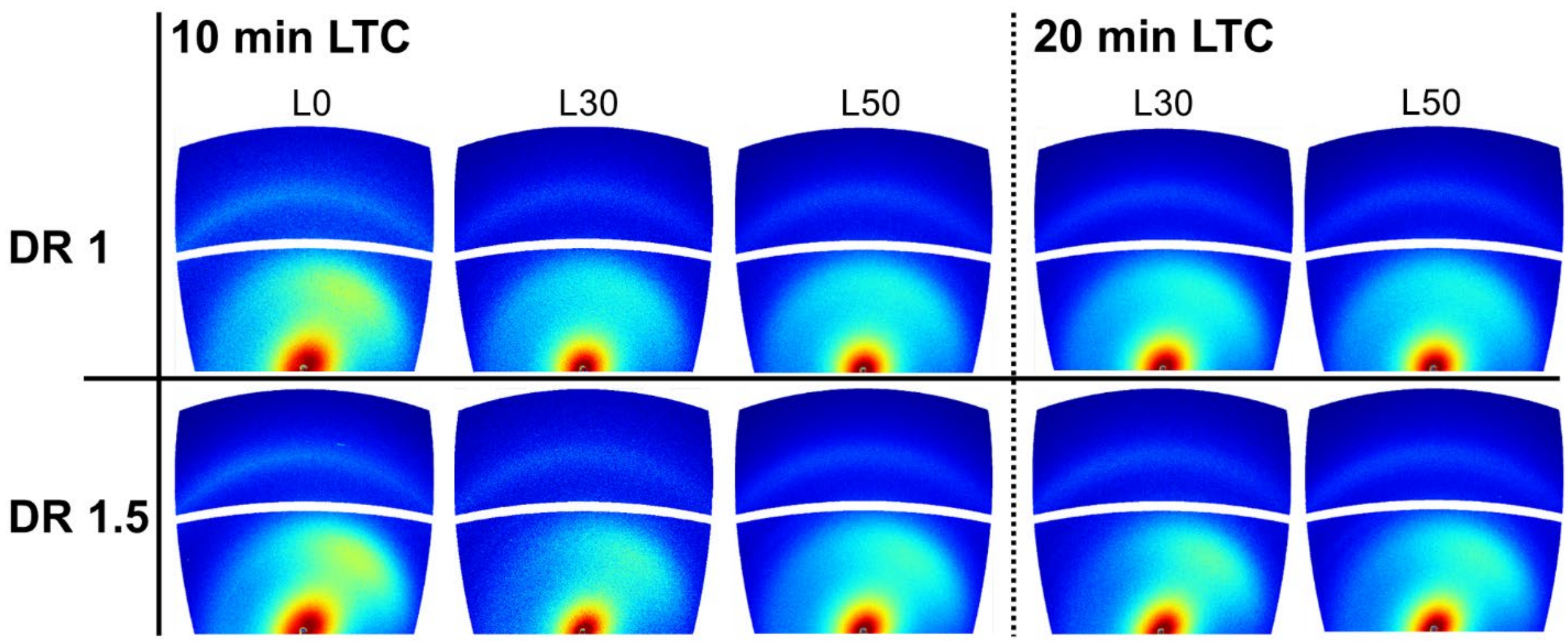

Figure S5. 2D WAXS images of CFs prepared from the different precursor filaments using DR 1.0 or 1.5 during LTC (850 °C, 10 or 20 min). Stabilization profile 245 °C for 30 min using DR 1.0.

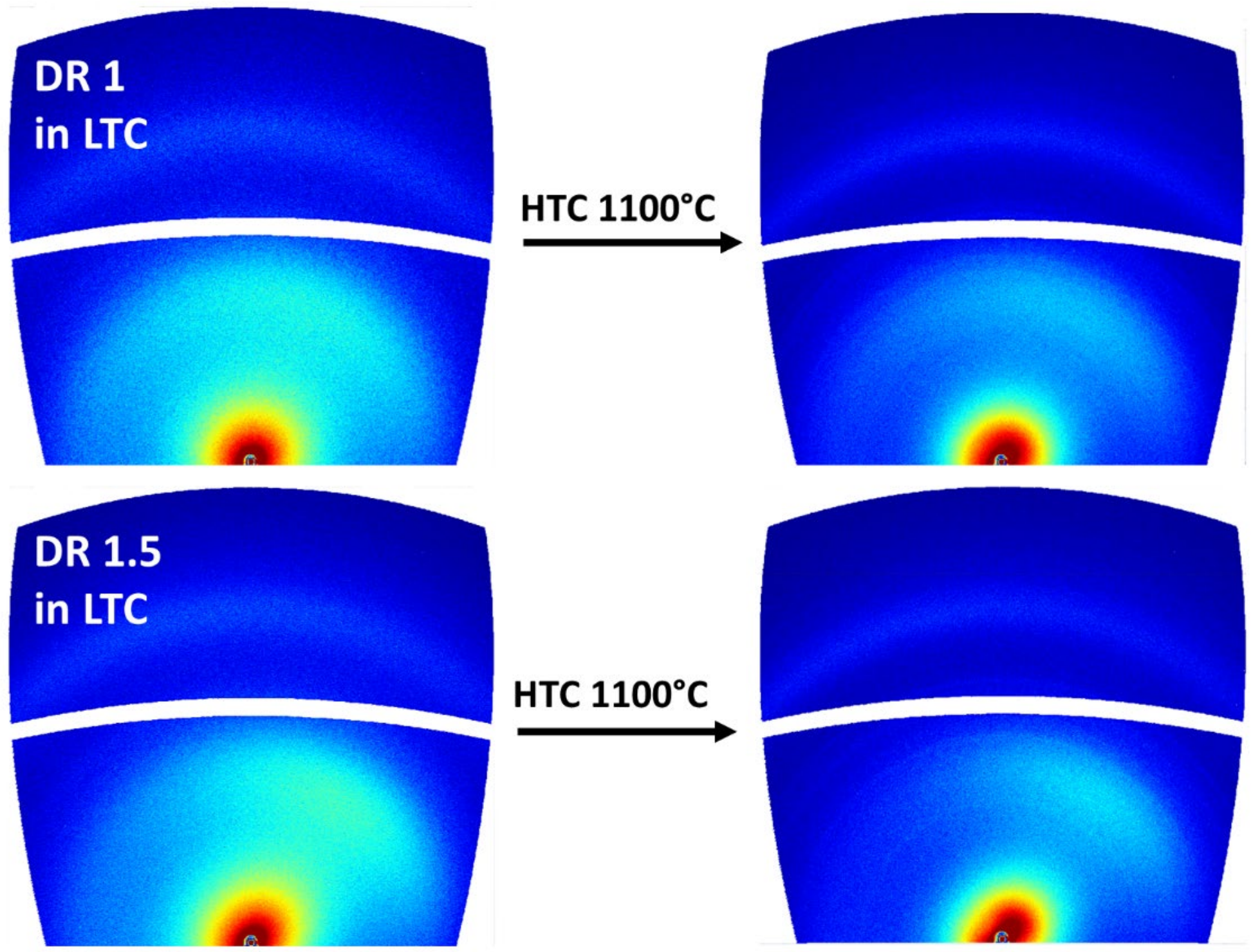

Figure S6. 2D WAXS images of LTC and HTC L50 fibers prepared from the different DR 1.0 or 1.5 during LTC (850 °C, 10 min).

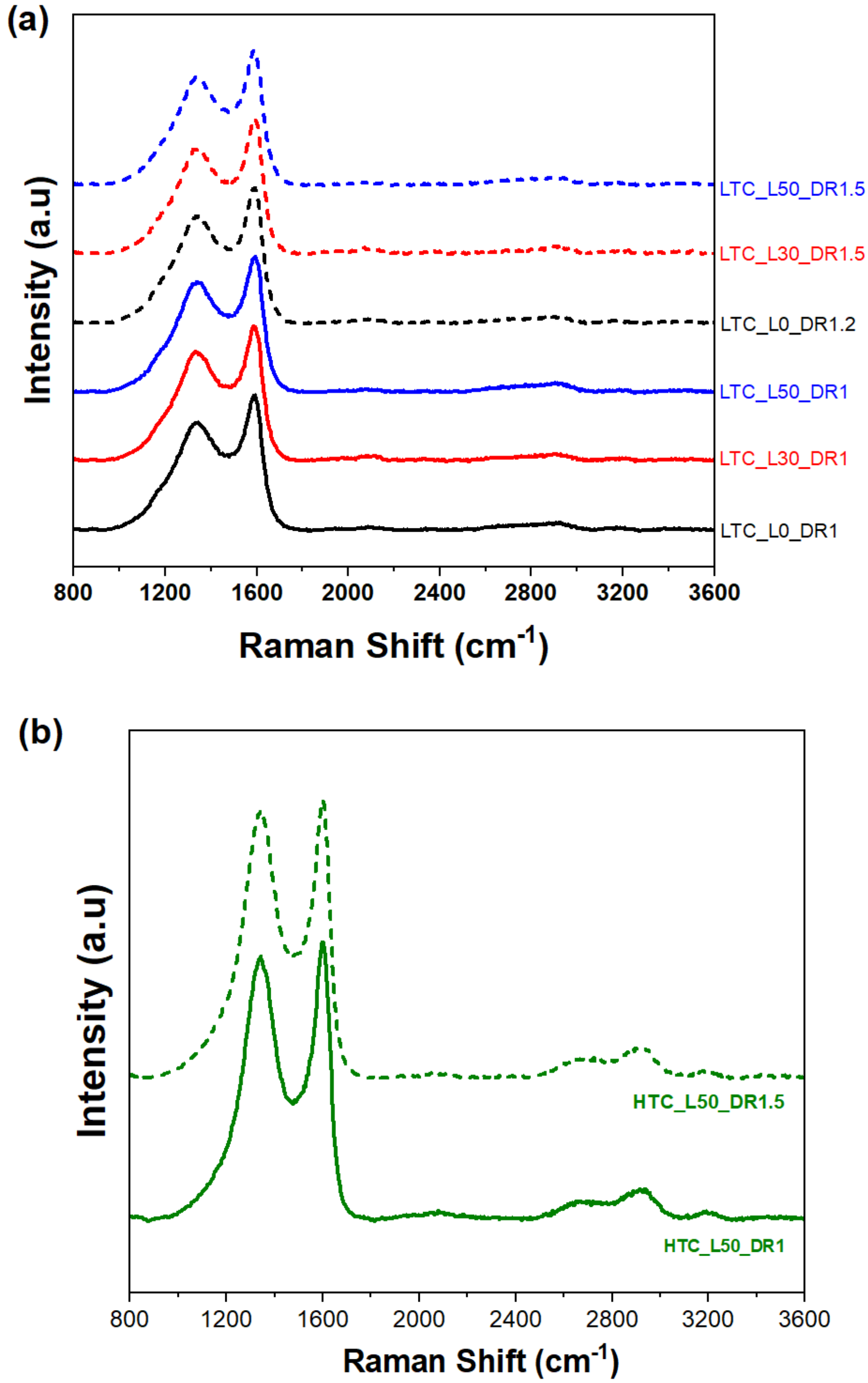

Figure S7. Raman spectra of cellulose-SKL derived CFs prepared at different carbonization temperature and draw ratio (DR1.0 or DR1.5) during LTC (a) LTC: 850 °C for 10 min; (b) HTC: 1100 °C for 10 min.

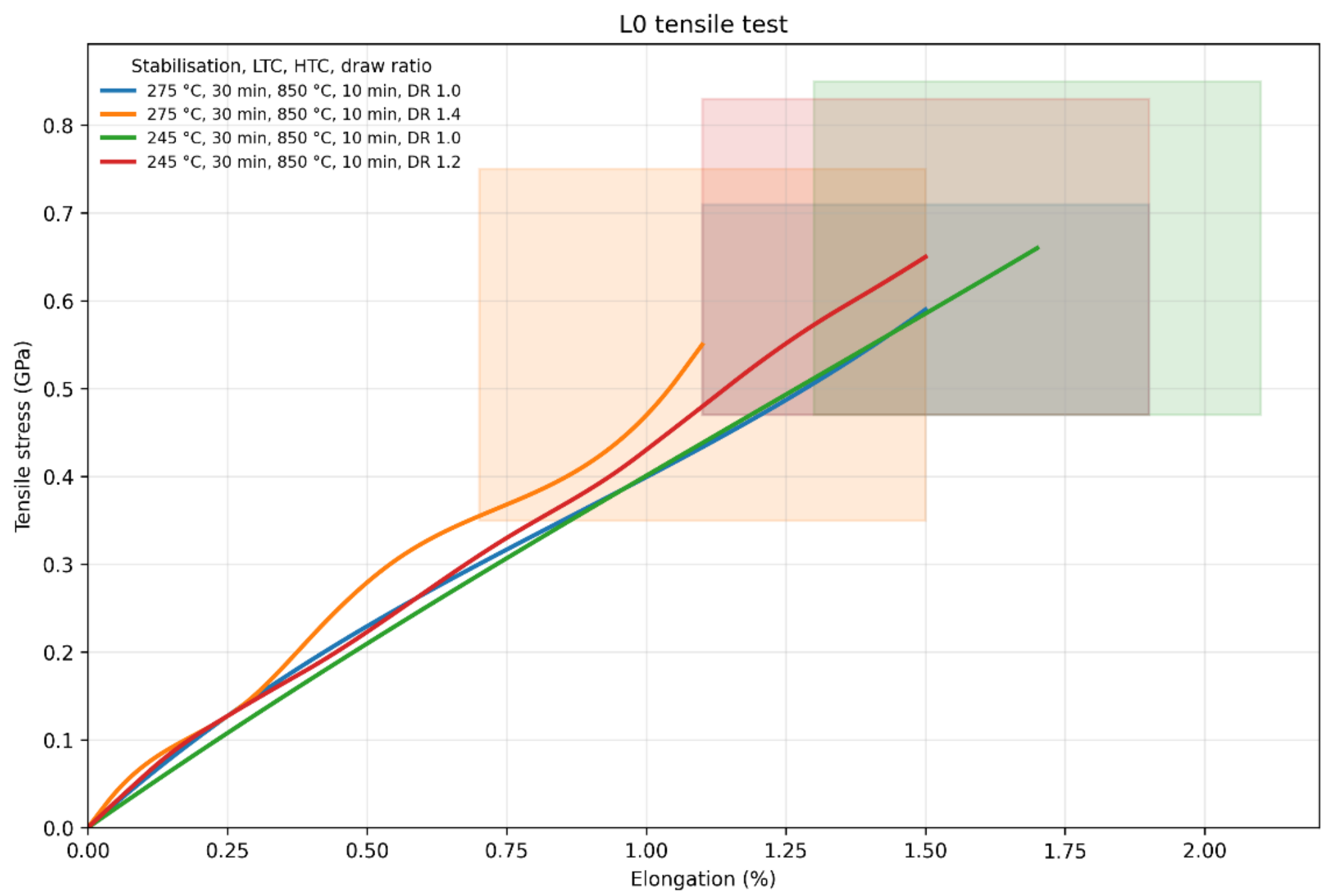

Figure S8. Stress-strain curves for the different L0 carbon fibers.

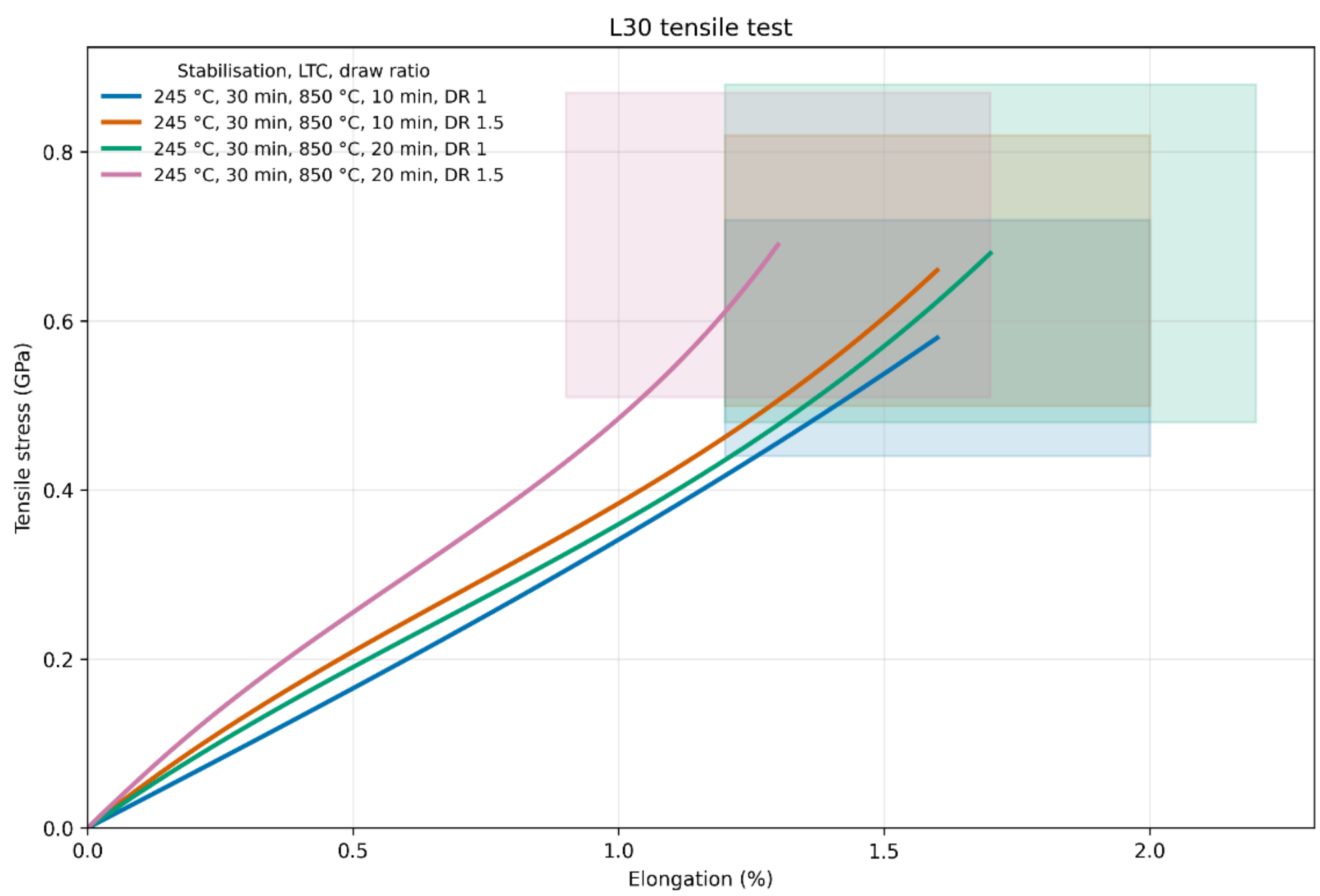

Figure S9. Stress-strain curves for the different L30 carbon fibers.

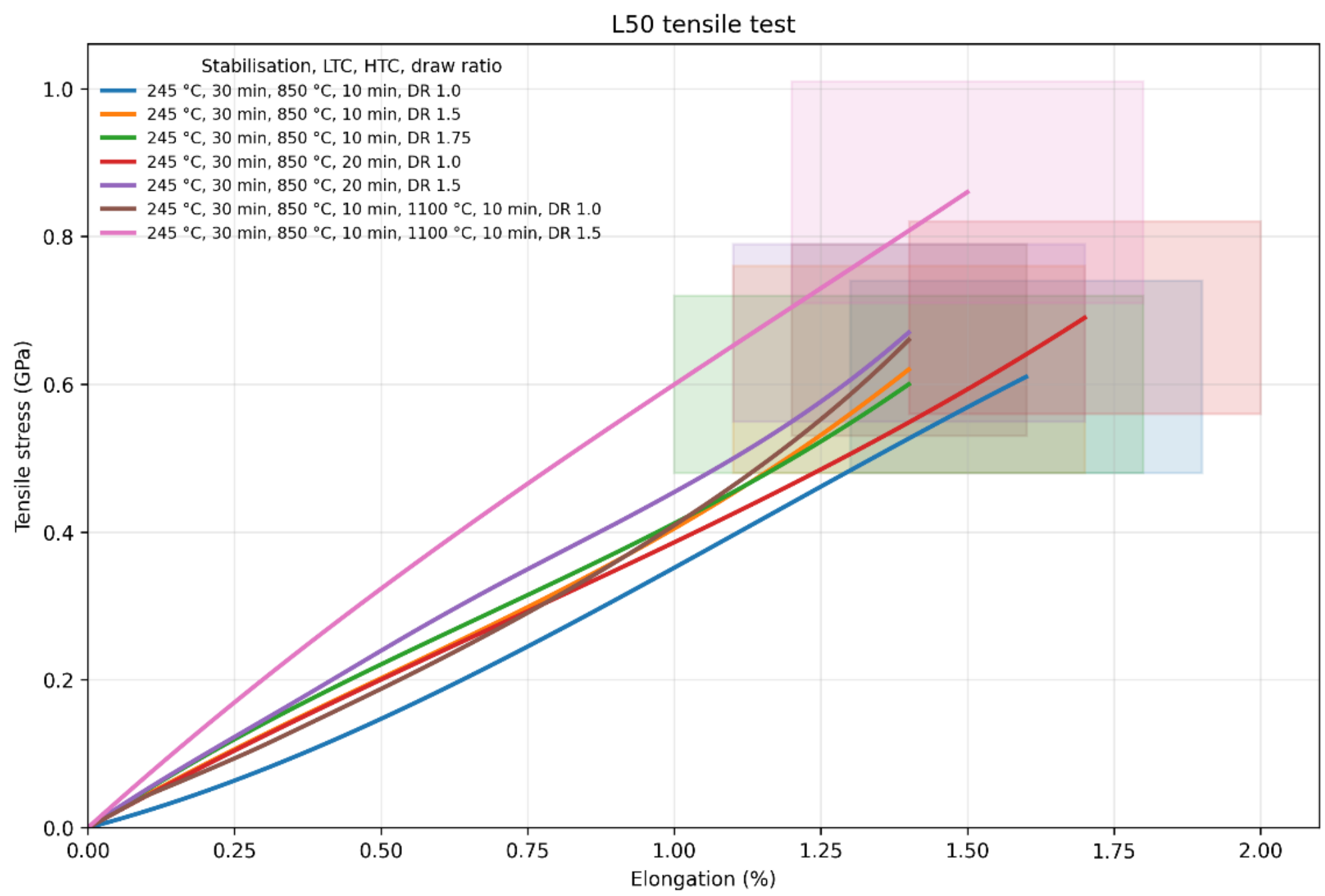

Figure S10. Stress-strain curves for the different L50 carbon fibers.

Table S1. Effect of precursor, stabilization and LTC conditions on DR_{max}.

Precursor fiber	Stabilization temperature (°C)	Stabilization time (min)	LTC temperature (°C)	LTC time (min)	DR_{max}
L0	-	-	850	10	1.4
L0	245	30	850	10	1.4
L0	245	30	850	20	1.4
L0	275	30	850	10	1.7
L30	-	-	700	10	2.3
L30	245	30	700	10	2.4
L30	245	60	700	10	2.0
L30	275	30	700	10	1.8
L30	245	30	850	10	2.0
L30	245	30	850	20	1.7
L50	-	-	850	10	2.1
L50	245	30	850	10	2.0
L50	245	30	850	20	1.7
L50	275	30	850	10	1.5

Table S2. Tensile properties of the carbon fibers obtained at various conditions (standard deviation in parentheses).

Precursor fiber	Stabilization/LTC/HTC			Young's modulus (GPa)	Tensile strength (GPa)	Elongation at break (%)	Diameter (μm)	n
	Temperature (°C)	Time (min)	DR					
L0	275/850/-	30/10/-	1.0/1.0/-	41 (6.8)	0.59 (0.12)	1.5 (0.4)	4.3 (0.3)	19
L0	275/850/-	30/10/-	1.0/1.4/-	47 (14.5)	0.55 (0.20)	1.1 (0.4)	3.8 (0.1)	13
L0	245/850/-	30/10/-	1.0/1.0/-	41 (6.3)	0.66 (0.19)	1.7 (0.4)	4.4 (0.4)	24
L0	245/850/-	30/10/-	1.0/1.2/-	46 (7.0)	0.65 (0.18)	1.5 (0.4)	4.0 (0.5)	22
L30	245/850/-	30/10/-	1.0/1.0/-	36 (2.9)	0.58 (0.14)	1.6 (0.4)	6.2 (0.3)	15
L30	245/850/-	30/10/-	1.0/1.5/-	45 (6.3)	0.66 (0.16)	1.6 (0.4)	5.0 (0.4)	23
L30	245/850/-	30/20/-	1.0/1.0/-	41 (3.7)	0.68 (0.20)	1.7 (0.5)	6.0 (0.6)	19
L30	245/850/-	30/20/-	1.0/1.5/-	54 (8.4)	0.69 (0.18)	1.3 (0.4)	5.0 (0.4)	24
L50	245/850/-	30/10/-	1.0/1.0/-	38 (3.3)	0.61 (0.13)	1.6 (0.3)	7.7 (0.4)	23
L50	245/850/-	30/10/-	1.0/1.5/-	44 (3.2)	0.62 (0.14)	1.4 (0.3)	6.3 (0.5)	25
L50	245/850/-	30/10/-	1.0/1.75/-	44 (5.2)	0.60 (0.12)	1.4 (0.4)	5.9 (0.5)	28
L50	245/850/-	30/20/-	1.0/1.0/-	41 (3.8)	0.69 (0.13)	1.7 (0.3)	7.8 (0.5)	32
L50	245/850/-	30/20/-	1.0/1.5/-	49 (5.2)	0.67 (0.12)	1.4 (0.3)	6.2 (0.4)	32
L50	245/850/1100	30/10/10	1.0/1.0/0.98	46 (3.7)	0.66 (0.13)	1.4 (0.2)	7.3 (0.4)	31
L50	245/850/1100	30/10/10	1.0/1.5/0.98	58 (4.7)	0.86 (0.15)	1.5 (0.3)	6.0 (0.3)	36

Table S3. Yield and elemental composition of the carbon fibers.

Precursor fiber	Stabilization/LTC/HTC			Yield (%)	C (wt%)	O (wt%)	S (wt%)
	Temperature (°C)	Time (min)	DR				
L0	-/850/-	-/10/-	-/1.4/-	7	-	-	-
L0	275/850/-	30/10/-	1.0/1.0/-	-	89	11	0
L0	275/850/-	30/10/-	1.0/1.4/-	-	89	11	0
L0	245/850/-	30/10/-	1.0/1.0/-	7	87	12	0
L0	245/850/-	30/10/-	1.0/1.2/-	7	88	12	0
L30	245/850/-	30/10/-	1.0/1.0/-	21	88	10	0.5
L30	245/850/-	30/10/-	1.0/1.5/-	19	88	8	0.7
L30	245/850/-	30/20/-	1.0/1.0/-	21	87	11	0.3
L30	245/850/-	30/20/-	1.0/1.5/-	19	-	-	-
L50	245/850/-	30/10/-	1.0/1.0/-	28	87	11	0.3
L50	245/850/-	30/10/-	1.0/1.5/-	28	89	8	0.8
L50	245/850/-	30/10/-	1.0/1.75/-	26	-	-	-
L50	245/850/-	30/20/-	1.0/1.0/-	29	-	-	-
L50	245/850/-	30/20/-	1.0/1.5/-	30	88	8	1.0
L50	245/850/1100	30/10/10	1.0/1.0/0.98	27	91	9	0.7
L50	245/850/1100	30/10/10	1.0/1.5/0.98	27	-	-	-

Table S4. The preferred orientation of 002-plane from WAXS and the intensity ratio (I_D/I_G) and FWHM of D- and G-bands from Raman.

Precursor fiber	Stabilization/LTC/HTC			Preferred orientation 002-plane	I_D/I_G	$FWHM_D$ (cm^{-1})	$FWHM_G$ (cm^{-1})
	Temperature (°C)	Time (min)	DR				
L0	275/850/-	30/10/-	1.0/1.0/-	0.60	0.80	233	84
L0	275/850/-	30/10/-	1.0/1.4/-	0.64	0.79	240	84
L0	245/850/-	30/10/-	1.0/1.0/-	0.60	0.79	238	85
L0	245/850/-	30/10/-	1.0/1.2/-	0.62	0.78	237	84
L30	245/850/-	30/10/-	1.0/1.0/-	0.49	0.80	230	86
L30	245/850/-	30/10/-	1.0/1.5/-	0.62	0.77	229	83
L30	245/850/-	30/20/-	1.0/1.0/-	0.54	0.83	234	91
L30	245/850/-	30/20/-	1.0/1.5/-	0.62	0.82	239	89
L50	245/850/-	30/10/-	1.0/1.0/-	0.43	0.80	231	87
L50	245/850/-	30/10/-	1.0/1.5/-	0.58	0.79	235	86
L50	245/850/-	30/10/-	1.0/1.75/-	0.60	0.78	229	84
L50	245/850/-	30/20/-	1.0/1.0/-	0.47	0.83	232	89
L50	245/850/-	30/20/-	1.0/1.5/-	0.60	0.83	239	90
L50	245/850/1100	30/10/10	1.0/1.0/0.98	0.51	0.92	172	74
L50	245/850/1100	30/10/10	1.0/1.5/0.98	0.60	0.93	177	75

Table S5. Properties of Cellulose and Cellulose-Lignin-Based Carbon Fibers**.**

Precursor Type	Carbonization Processing Conditions	Tensile Strength (GPa)	Young's Modulus (GPa)	Carbon Yield (wt%)	Diameter (µm)	Ref
**Cellulose (Rayon/Viscose)**						
Rayon (Thornel 50-100)	Hot Stretching > 2600 °C	4.0	690	10–20	—	1
Rayon (Standard)		0.5–1.2	40–100	10–30	—	
Commercial cellulosic Tire cord	Continuous, 1400 °C	2.8	100	—	11	2
High-Strength Viscose	Optimized conversion	2.0	84	37	—	3
**Cellulose (Lyocell/Ioncell)**						
Lyocell (commercial)	No active stretch	0.5–0.9	40–90	—	—	4,5
Hardwood pulp Wet-jet spun	Continuous, 1400 °C	0.84-1.4	65-70	—	2.5	5
Softwood pulp Air gap spun	Continuous, 1400 °C	1.8	112	—	—	2
**Lignin-Cellulose Blends**						
30% Hardwood Lignin / 70%Dissolving pulp	Continuous, 1500 °C	0.66-0.72	59-66	—	8.2-8.8	6
	Continuous, 1950 °C	0.43-0.61	84-114	—	7.7-8.5	6
50% Soft wood lignin / 50% dissolving Pulp	Continuous, 1000 °C	0.71–0.92	46–51	29–30	12–15	7
	Batch, 1000 °C	0.80–0.92	63–67	34	12–15	7
70% soft wood lignin / 30% soft wood pulp	Batch, 1000 °C	0.88–1.05	64–67	~40	14–15	8
	Batch, 1600 °C	1.03–1.17	67–77	—	6.4–7.6	8
70% soft wood lignin / 30% soft wood pulp	Batch, 1000 °C flame retardant	~1.07	76	46–50	7.3-7.6	9
10–30% Softwood lignin/softwood dissolving pulp	Continuous,1100 °C	0.48–1.17	33–46	12–44	6–13	10

**This work**						
**100% cellulose**	Continuous Stretching at LTC 850 °C	0.55	47	7	3.8	
**30% lignin 70% cellulose**	Continuous Stretching at LTC ,850 °C	0.69	54	19-21	5	**This work**
**50% lignin 50% cellulose**	Continuous Stretching at LTC ,1100 °C	0.86	58	27	6	

References

(1) Zhang, X.; Lu, Y.; Xiao, H.; Peterlik, H. Effect of Hot Stretching Graphitization on the Structure and Mechanical Properties of Rayon-Based Carbon Fibers. *J. Mater. Sci.* **2014**, *49* (2), 673–684 DOI:10.1007/s10853-013-7748-0.

(2) Vocht, M. P.; Ota, A.; Frank, E.; Hermanutz, F.; Buchmeiser, M. R. Preparation of Cellulose-Derived Carbon Fibers Using a New Reduced-Pressure Stabilization Method. *Ind. Eng. Chem. Res.* **2022**, *61* (15), 5191–5201 DOI:10.1021/acs.iecr.2c00265.

(3) Spörl, J. M.; Beyer, R.; Abels, F.; Cwik, T.; Müller, A.; Hermanutz, F.; Buchmeiser, M. R. Cellulose-Derived Carbon Fibers with Improved Carbon Yield and Mechanical Properties. *Macromol. Mater. Eng.* **2017**, *302* (10) DOI:10.1002/mame.201700195.

(4) Dumanlı, A. G.; Windle, A. H. Carbon Fibres from Cellulosic Precursors: A Review. *Journal of Materials Science*. Kluwer Academic Publishers 2012, pp 4236–4250 DOI:10.1007/s10853-011-6081-8.

(5) Byrne, N.; Setty, M.; Blight, S.; Tadros, R.; Ma, Y.; Sixta, H.; Hummel, M. Cellulose-Derived Carbon Fibers Produced via a Continuous Carbonization Process: Investigating Precursor Choice and Carbonization Conditions. *Macromol. Chem. Phys.* **2016**, *217* (22), 2517–2524 DOI:10.1002/macp.201600236.

(6) Unterweger, C.; Schlapp-Hackl, I.; Fürst, C.; Robertson, D.; Cho, M. J.; Hummel, M. Carbon Fibers Based on Cellulose–Lignin Hybrid Filaments: Role of Dehydration Catalyst, Temperature, and Tension during Continuous Stabilization and Carbonization. *Fibers* **2024**, *12* (7) DOI:10.3390/fib12070055.

(7) Bengtsson, A.; Bengtsson, J.; Jedvert, K.; Kakkonen, M.; Tanhuanpää, O.; Brännvall, E.; Sedin, M. Continuous Stabilization and Carbonization of a Lignin-Cellulose Precursor to Carbon Fiber. *ACS Omega* **2022** DOI:10.1021/acsomega.2c01806.

(8) Bengtsson, A.; Hecht, P.; Sommertune, J.; Ek, M.; Sedin, M.; Sjöholm, E. Carbon Fibers from Lignin–Cellulose Precursors: Effect of Carbonization Conditions. *ACS Sustain. Chem. Eng.* **2020**, *8* (17), 6826–6833 DOI:10.1021/acssuschemeng.0c01734.

(9) Bengtsson, A.; Bengtsson, J.; Sedin, M.; Sjöholm, E. Carbon Fibers from Lignin-Cellulose Precursors: Effect of Stabilization Conditions. *ACS Sustain. Chem. Eng.* **2019**, *7* (9), 8440–8448 DOI:10.1021/acssuschemeng.9b00108.

(10) Bengtsson, A.; Landmér, A.; Norberg, L.; Yu, S.; Ek, M.; Brännvall, E.; Sedin, M. Carbon Fibers from Wet-Spun Cellulose-Lignin Precursors Using the Cold Alkali Process. *Fibers* **2022**, *10* (12) DOI:10.3390/fib10120108.
